# Long-Term Outcomes of COVID-19 Otolaryngology Symptoms in Saudi Arabia

**DOI:** 10.7759/cureus.33461

**Published:** 2023-01-06

**Authors:** Musaed Alzahrani, Almaha H Alshathri, Khalil Alduraibi, Aljohara H Alshathri, Tahani F Alanazi, Hamza Alandijani, Jumana a Almajed, Khloud A Wajdi

**Affiliations:** 1 Department of Otolaryngology - Head and Neck Surgery, King Fahad Specialist Hospital, Dammam, SAU; 2 College of Medicine, King Saud University, Riyadh, SAU; 3 Department of General Medicine, College of Medicine and Surgery, King Saud University, Riyadh, SAU; 4 Department of Otolaryngology - Head and Neck Surgery, University of Tabuk, Tabuk, SAU; 5 College of Medicine, Taibah University, Almadinah Almunawwarah, SAU; 6 Department of Otolaryngology - Head and Neck Surgery, King Abdulaziz University, Jeddah, SAU; 7 Department of Pharmacology, Ibn Sina National College, Jeddah, SAU

**Keywords:** otology, vertigo, covid- 19, orl, long-term complications

## Abstract

Objectives: The objectives of the study are to measure the prevalence of the most common symptoms and different long-term otorhinolaryngology manifestations among COVID-19-positive patients in Saudi Arabia.

Methods: This is a cross-sectional study. Data were collected using a Google form questionnaire sent to the study sample. The data were entered and then analyzed using SPSS version 25.0 (IBM Corp, Armonk, NY).

Results: A total of 13,530 COVID-19-positive adult patients were enrolled in the current study. The most reported initial symptom was fever (53.3%) followed by headache (49.3%), sore throat (48.9%), nasal congestion, rhinorrhea (42.1%), and loss of smell and/or loss of taste (37.8%). Regarding the continuation of the symptoms, vertigo was mentioned by 5852 (43.3%) followed by fever (16.1%) and blocked ear sensation (15.6%).

Conclusion: The most commonly reported initial symptom was fever followed by headache. Interestingly, vertigo is a frequent long-term complication after a COVID-19 infection. Other otology-related symptoms include hearing loss and ear blockage, while rhinology and upper airway-related symptoms were less frequent in the long term after the acute phase of the infection.

## Introduction

COVID-19 is an infectious disease that started in Wuhan, China, and the onset of the first known case dates back to 8 December 2019. On 11 March 2020, the WHO officially characterized the global COVID-19 outbreak as a pandemic [1]. Several studies showed that common symptoms are fever, fatigue, and dry cough [1-4]. However, a study by Telmesani et al. conducted through three centers in various regions of Saudi Arabia revealed that the initial symptoms of otorhinolaryngology (ORL) were sore throat, anosmia, and loss of taste [5].

Some patients suffer from long COVID-19 or post-COVID-19 conditions, which are defined as a wide range of symptoms lasting more than four weeks or even months after infection [6]. Multiple studies reported long-term COVID-19 effects on different systems including respiratory, cardiovascular, gastrointestinal, neurological, and psychiatric manifestations [7,8]. Two of these studies revealed that the most common persistent symptoms were fatigue and weakness followed by headache [8,9]. AlRadini et al. showed that the most common persistent symptoms are loss of smell, loss of taste, fatigue, shortness of breath, and cough in patients with post-acute COVID-19 conditions [10]. Another study showed that more than half of the patients had persistent symptoms including loss of smell, loss of taste, cough, and fatigue [11]. A recent study applied to the Saudi population found that female gender, old age, number of chronic complications, long-term medication, length of hospital stays, intensive care unit admission, and duration of acute symptoms may be significant predictors of post-COVID-19 symptoms [12].

A systematic review of 23 studies about the long-term outcome of COVID-19 found persistent respiratory symptoms, fatigue, decreased functional capacity, and decreased quality of life up to six months after infection [13]. Moreover, a systematic review of 145 studies showed that 20.7% of reports on long-term COVID-19 symptoms were on abnormal lung functions, 24.13% on neurologic complaints and olfactory dysfunctions, and 55.17% on specific widespread symptoms, mainly chronic fatigue and pain [14]. These studies highlight the importance to look for ORL specifically.

We aim in this study to measure the prevalence of the most common ORL symptoms among COVID-19-positive patients in Saudi Arabia. We also aim from this study to investigate and discuss different long-term ORL manifestations in those who were reported as COVID-19-positive adult patients. 

## Materials and methods

This is a cross-sectional study. Data were collected from August to September 2022, and the study targeted those who were infected with COVID-19 from the beginning of the disease in Saudi Arabia. An online questionnaire designed on Google Forms was used as a tool for the study and was sent to the study sample.

The questionnaire consists of sections, and each section contains a set of questions. The first section includes the demographic characteristics of the participants. The second section includes clinical data, such as initial presentation, duration, general symptoms during the acute phase of the disease, and long-term symptoms that appeared or continued from the acute phase for a month at least. While the third, fourth, and fifth sections detailed the rhinology, upper airway/gastroesophageal reflux disease (GERD), and otology findings, respectively. They were measured by the 4-point Likert scale, where 1 indicates “never,” 2 indicates “sometimes,” 3 indicates “often,” and 4 indicates “always.” This section was added to evaluate the impact of each symptom on the participants.

All data were entered into the analytical program V.25 of the Statistical Package for the Social Sciences (SPSS; IBM Corp, Armonk, NY). Descriptive analysis was used for demographic and clinical data sections. Mann-Whitney test was used to study the differences between smokers and non-smokers in relation to symptoms of COVID-19 infections. Finally, the chi-square (χ^2^) test was used to find out the relationship between smoking and other factors, and a P-value ≤0.05 was considered statistically significant.

We obtained approval for this work through the Ethics Committee of King Fahad Specialist Hospital - Dammam (approval number: ENT0004). 

## Results

Background and demographic information 

A total of 13,530 COVID-19-positive adult patients were included in this study. Official data from the Ministry of Health reported a total of 817,838 COVID-19-confirmed cases in Saudi Arabia and 9369 mortality cases. The study sample represents 2% of the study population. Most of them were females (68.5%) while 4261 (31.5%) were males. The majority of them were in the age group of 18-25 and 26-40, which represent 48.3% and 31.5%, respectively. The majority were of Saudi nationality (88.1%) while 1605 (11.9%) non-Saudi. A total of 11,347 (83.9%) were non-smokers (83.9%) and 2183 (16.1%) were smokers. Regarding other comorbidities, most of the study participants had no disease other than COVID-19 (63.1%), while 1567 had diabetes (11.6%), 1477 had asthma (10.9%), 1246 had morbid obesity (9.2%), 1153 had hypertension (8.5%), and 566 (4.2%) had other illnesses. Most of the study participants did not have a history of ORL diseases (63.1%) before the infection while 3603 (26.6%) had a history of allergic rhinosinusitis. In more than one-third of the participants, 12 months have passed since they were diagnosed as having infection with COVID-19. Concerning the source of COVID-19 infection, close contact with the known case was the most reported source (52.2%) followed by healthcare workers (27.5%). The majority of the participants were treated by home isolation (93.8%). Finally, 11,396 (84.2%) recovered without complications, 1852 (13.7%) recovered but still have complications, 169 (1.3%) have not recovered yet with symptoms improvement, and 60 (0.4%) have not recovered yet and their symptoms have worsened (Table 1).

**Table 1:** Sociodemographic and clinical information of the participants (n = 13,530)

Variables	Categories	N (%)
Gender	Female	9269 (68.5)
	Male	4261 (31.5)
Age (years)	18-25	6533 (48.3)
	26-40	4266 (31.5)
	41-60	2423 (17.9)
	60 and above	308 (2.3)
Nationality	Saudi	11,925 (88.1)
	Non-Saudi	1605 (11.9)
Are you a smoker?	No	11,347 (83.9)
	Yes	2183 (16.1)
Comorbidity	Asthma	1477 (10.9)
	Hypertension	1153 (8.5)
	Diabetes	1567 (11.6)
	Obesity	1246 (9.2)
	None	9232 (68.2)
	Other	566 (4.2)
Do you have a history of any otorhinolaryngological diseases?	Allergic rhinosinusitis	3603 (26.6)
	Chronic sinusitis	720 (5.3)
	Hearing loss	593 (4.4)
	Vertigo	1070 (7.9)
	None	8533 (63.1)
	Other	62 (0.5)
How many months have passed since you have been diagnosed with COVID-19?	3 months	3117 (23)
	6 months	3141 (23.2)
	9 months	1890 (14)
	12 months	243 (1.8)
	More than 12 months	4693 (34.7)
	Other	446 (3.3)
What is the source of infection of COVID-19?	Close contact with known case	7068 (52.2)
	Occupational exposure	1466 (10.8)
	Travel	868 (6.4)
	Healthcare worker	3722 (27.5)
	Unidentifiable	398 (2.9)
	Other	8 (0.06)
How was your COVID-19 treated?	Home isolation	12,693 (93.8)
	Hospital admission	654 (4.8)
	ICU	183 (1.4)
What is your current condition?	Recovered from COVID-19 without complications	11,396 (84.2)
	Recovered from COVID-19 but I still have complications	1852 (13.7)
	Symptoms improved but no recovery yet	169 (1.3)
	Symptoms worsened and no recovery yet	60 (0.4)
	Missing data	53 (0.4)

Clinical presentation

Regarding the clinical presentation of the participating patients, the most reported initial symptom was fever (53.3%) followed by headache (49.3%), sore throat (48.9%), nasal congestion, rhinorrhea (42.1%), and loss of smell and/or loss of taste (37.8%).

Initial symptoms resolved in less than a week in more than half of the participants (52.8%), between a week to a month in 5568 (41.2%), and in more than a month in 817 (6%). General symptoms during the acute phase of the infection are detailed in Table 2. When the participants asked about the appearance of symptoms or the continuation after COVID-19 treatment, vertigo was reported by 5852 (43.3%) of the participants followed by fever (16.1%) and blocked ear sensation (15.6%).

**Table 2:** Initial symptoms, general and otorhinolaryngology symptoms associated with COVID-19 infection

Questions	Categories	Frequency (%)
What are the initial presenting symptoms when you were diagnosed with COVID-19?	Anosmia and/or loss of taste	5113 (37.8)
	Nasal congestion and rhinorrhea	5690 (42.1)
	Sore throat	6613 (48.9)
	Fever	7206 (53.3)
	Hearing loss	784 (5.8)
	Headache	6674 (49.3)
	Vertigo	2310 (17.1)
	No symptoms (discovered with laboratory test)	1633 (12.1)
For how long did your symptoms last?	Less than 1 week	7145 (52.8)
	1 week-1 month	5568 (41.2)
	More than 1 month	817 (6)
What are the general symptoms did you report when you were diagnosed with COVID-19?	Asymptomatic	1591 (11.8)
	Fever	8080 (59.7)
	Cough/shortness of breath	6532 (48.3)
	Headache	7615 (56.3)
	Myalgia/arthralgia	6896 (51)
	Nausea or vomiting	2226 (16.5)
	Abdominal pain	1801 (13.3)
	Diarrhea	2185 (16.2)
	Nasal congestion/rhinorrhea	5485 (40.5)
	Sore throat	5706 (42.2)
	Hearing loss	701 (5.2)
	Blocked ear sensation	2481 (18.3)
	Vertigo	1303 (9.6)
	Tinnitus	5098 (37.7)
Did you any of the following symptoms appeared/or continued after COVID-19 treatment?	Asymptomatic	940 (7)
	Fever	2183 (16.1)
	Cough/shortness of breath	1847 (13.7)
	Headache	1824 (13.5)
	Myalgia/arthralgia	582 (4.3)
	Nausea or vomiting	500 (3.7)
	Abdominal pain	1062 (7.9)
	Diarrhea	768 (5.7)
	Nasal congestion/rhinorrhea	525 (3.9)
	Sore throat	746 (5.5)
	Hearing loss	655 (4.8)
	Blocked ear sensation	2110 (15.6)
	Vertigo	5852 (43.3)

Characterization of rhinology symptoms, upper airway/GERD-related symptoms, and otology-related symptoms of COVID-19

Measuring the reliability of the scale for these three sections was done by calculating the value of Cronbach’s alpha. It reached 0.727, 0.899, and 0.836 on the third, fourth, and fifth sections scale, respectively, which means it was reliable.

This section detailed the rhinology symptoms: 7599 (56.2%) suffered nasal congestion/rhinorrhea with varying degrees. More than half of the participants, 7377 (54.5%), have never had anosmia/hyposmia. About 8263 (61.1%) of the participants have never been exposed to postnasal drip. Likewise, the majority did not complain of facial pain/pressure or epistaxis, 9796 (72.4%) and 11595 (85.7%), respectively. 

Concerning the upper airway/GERD symptoms, sore throat was the only symptom experienced by more than half the population, 7301 (54%), while loss of taste, dysphagia, hoarseness of voice, and heartburn were positive in 6108 (45%), 5221 (38.6%), 5715 (42.2%), and 3734 (27.5%), respectively. 

As for otology symptoms, dizziness and vertigo were the most prevalent among other symptoms with an incidence of 5266 (38.9%). Other symptoms were less frequent, ear pain/pressure 3527 (26%), tinnitus 3294 (24.3%), hearing loss 1678 (12.4%), and ear fullness 2964 (21.9%) (Table 3).

**Table 3:** Characterization of rhinology symptoms, upper airway/GERD-related symptoms and otology-related symptoms during the patient's diagnosis of COVID-19 GERD, gastroesophageal reflux disease.

Symptoms	Never	Sometimes	Usually	Always
	N (%)			
Rhinology symptoms				
Nasal congestion/rhinorrhea	5931 (43.8)	3970 (29.3)	1904 (14.1)	1725 (12.7)
Anosmia/hyposmia	7377 (54.5)	2689 (19.9)	1600 (11.8)	1864 (13.8)
Postnasal drip	8263 (61.1)	2923 (21.6)	1397 (10.3)	947 (7)
Facial pain/pressure	9797 (72.4)	2227 (16.5)	914 (6.8)	592 (4.4)
Epistaxis	11,595 (85.7)	1207 (8.9)	443 (3.3)	285 (2.1)
Upper airway/GERD symptoms				
Sore throat	6229 (46)	3373 (24.9)	1767 (13.1)	2161 (16)
Loss of taste	7422 (54.9)	2521 (18.6)	1556 (11.5)	2031 (15)
Dysphagia	8309 (61.4)	2780 (20.5)	1296 (9.6)	1145 (8.5)
Hoarseness of voice	7815 (57.8)	2852 (21.1)	1412 (10.4)	1451 (10.7)
Heartburn	9796 (72.4)	2269 (16.8)	796 (5.9)	669 (4.9)
Otology-related symptoms				
Ear pain/pressure	10,003 (73.9)	2193 (16.2)	665 (4.9)	669 (4.9)
Tinnitus	10,236 (75.7)	2088 (15.4)	705 (5.2)	501 (3.7)
Hearing loss	11,852 (87.6)	1020 (7.5)	450 (3.3)	208 (1.5)
Dizziness/vertigo	8264 (61.1)	2896 (21.4)	1344 (9.9)	1026 (7.6)
Ear fullness	10,566 (78.1)	1753 (13)	668 (4.9)	543 (4)

Association between smoking and symptoms associated with COVID-19 infection

Regarding the rhinology symptoms, it was found that there were significant differences between smokers and non-smokers in the prevalence of nasal congestion/rhinorrhea, anosmia/hyposmia, and epistaxis. Smokers had a significantly higher incidence of epistaxis (P-value < 0.002) while non-smokers had a significantly higher incidence of nasal congestion/rhinorrhea and anosmia/hyposmia (P-value < 0.001, < 0.001, respectively). There were no differences in postnasal drip and facial pain/pressure between smokers and non-smokers (P-value = 0.053, 0.525, respectively).

Concerning upper airway/GERD-related symptoms, there were significant differences between smokers and non-smokers regarding sore throat, loss of taste, dysphagia, and hoarseness of voice and it was in favor of non-smokers (P-value < 0.001, < 0.001, 0.008, and < 0.001, respectively), while there were no significant differences in heartburn between them (P-value = 0.682).

As for otology-related symptoms, hearing loss was higher among smokers (P-value < 0.001) while dizziness/vertigo was higher among non-smokers (P-value < 0.002). There were no differences in ear pain/pressure, tinnitus, and ear fullness between smokers and non-smokers (P-value = 0.924, 0.752, and 0.118, respectively) (Table 4).

**Table 4:** Association between smoking and symptoms associated with COVID-19 infection GERD, gastroesophageal reflux disease.

Symptoms		Smoking				P-value
		Non-smokers (n = 11,347)		Smokers (n = 2183)		
		Mean	SD	Mean	SD	
Rhinology symptoms	Nasal congestion/rhinorrhea	1.99	1.045	1.81	1.019	<0.001
	Anosmia/hyposmia	1.87	1.096	1.74	1.061	<0.001
	Postnasal drip	1.64	0.928	1.60	0.918	0.053
	Facial pain/pressure	1.43	0.801	1.42	0.806	0.525
	Epistaxis	1.21	0.590	1.26	0.660	0.002
Upper airway/GERD-related symptoms	Sore throat	2.02	1.116	1.83	1.059	<0.001
	Loss of taste	1.88	1.124	1.78	1.070	<0.001
	Dysphagia	1.66	0.968	1.60	0.931	0.008
	Hoarseness of voice	1.76	1.030	1.65	0.970	<0.001
	Heartburn	1.43	0.812	1.44	0.813	0.682
Otology-related symptoms	Ear pain/pressure	1.41	0.799	1.41	0.792	0.924
	Tinnitus	1.37	0.747	1.37	0.753	0.752
	Hearing loss	1.18	0.542	1.25	0.633	<0.001
	Dizziness/vertigo	1.65	0.945	1.58	0.914	0.002
	Ear fullness	1.34	0.748	1.37	0.778	0.118

Association between the degrees of recovery from COVID-19 and symptoms associated with infection

Rhinology symptoms, upper airway/GERD-related symptoms, and otology-related symptoms were significantly different according to the current condition of the participants (P-value < 0.001). It was observed that congestion/rhinorrhea and anosmia/hyposmia were higher among recovered participants who still have complications (P-value < 0.001), while postnasal drip, facial pain/pressure, and epistaxis were higher among the group of patients whose symptoms worsened but there was no recovery yet (P-value < 0.001).

Concerning upper airway/GERD-related symptoms, sore throat, loss of taste, and hoarseness of voice were higher among the participants who had recovered but still had complications (P-value < 0.001), while dysphagia and heartburn were higher among non-recovered participants with worsened symptoms (P-value < 0.001).

All the otology-related symptoms including ear pain/pressure, tinnitus, hearing loss, dizziness/vertigo, and ear fullness were found to be significantly higher among non-recovered participants with worsened symptoms (Table 5).

**Table 5:** Association between the degrees of recovery from COVID-19 and symptoms associated with infection GERD, gastroesophageal reflux disease.

Symptoms		Recovered without complications	Recovered but still have complications	Symptoms improved but no recovery yet	Symptoms worsened and no recovery yet	P-value
		Mean (SD)	Mean (SD)	Mean (SD)	Mean (SD)	
Rhinology symptoms	Nasal congestion/rhinorrhea	1.93 (1.04)	2.16 (1.07)	1.69 (0.99)	1.73 (1.06)	<0.001
	Anosmia/hyposmia	1.78 (1.06)	2.28 (1.19)	1.72 (0.85)	1.87 (1.07)	<0.001
	Postnasal drip	1.58 (0.89)	1.94 (1.06)	1.79 (0.91)	2.08 (1.08)	<0.001
	Facial pain/pressure	1.38 (0.75)	1.73 (0.98)	1.70 (0.87)	1.80 (1.01)	<0.001
	Epistaxis	1.19 (0.57)	1.34 (0.73)	1.61 (0.90)	1.73 (1.04)	<0.001
Upper airway/GERD-related symptoms	Sore throat	1.95 (1.10)	2.24 (1.13)	1.89 (1.09)	1.88 (1.08)	<0.001
	Loss of taste	1.80 (1.09)	2.28 (1.20)	1.76 (0.89)	1.93 (0.94)	<0.001
	Dysphagia	1.60 (0.94)	1.94 (1.07)	1.86 (0.92)	2.10 (1.02)	<0.001
	Hoarseness of voice	1.69 (1.00)	2.05 (1.12)	1.96 (1.01)	2.02 (1.08)	<0.001
	Heartburn	1.37 (0.75)	1.80 (1.04)	1.65 (0.86)	1.92 (1.01)	<0.001
Otology-related symptoms	Ear pain/pressure	1.34 (0.73)	1.79 (1.02)	1.75 (0.96)	2.00 (1.16)	<0.001
	Tinnitus	1.31 (0.68)	1.69 (0.98)	1.79 (0.89)	1.85 (1.02)	<0.001
	Hearing loss	1.15 (0.50)	1.37 (0.76)	1.59 (0.74)	1.87 (0.95)	<0.001
	Dizziness/vertigo	1.57 (0.89)	2.04 (1.09)	1.97 (0.97)	2.22 (1.14)	<0.001
	Ear fullness	1.28 (0.68)	1.70 (1.00)	1.67 (0.89)	1.93 (1.15)	<0.001

## Discussion

In this study, we have reported, of varying degrees, the long-term effects of COVID-19 on ORL symptoms. Otology symptoms were apparently most affected by long-term sequelae of COVID-19 infection. Vertigo was the most prevalent complaint with an interesting rate of 43.3%, while blocked ear sensation was reported by 15.6% of the participants. Hearing loss was only reported by 4.8%; however, it is worthy of consideration for future studies. General effects of COVID-19, such as fever, cough, and headache, were also reported with an incidence of less than 20%. Our result showed that rhinology symptoms were less frequent in the long term after the acute phase of the infection. Studying the long-term effects of COVID-19 infection on ORL is important as it could result in early prediction of these effects and hence prevention or reduction of undesirable symptoms' incidence through early suitable intervention and management.

Concerning comorbidities with COVID-19, diabetes was most reported by 11.6% of the participants followed by asthma (10.9%), and this was contradictory to Ng Wh et al.'s findings in which the most reported comorbidity among COVID-19 patients was hypertension followed by obesity [13].

As for the clinical presentation of the participating patients, the most commonly reported initial symptom when they were diagnosed with COVID-19 was fever (53.3%) followed by headache (49.3%), sore throat (48.9%), nasal congestion and rhinorrhea (42.1%), and loss of smell and/or loss of taste (37.8%). This was consistent with the findings reported by Amin et al. in which more than 50% of the participants experienced the previously mentioned symptoms as the initial presentation [14].

Regarding long-term symptoms after COVID-19 treatment, vertigo was reported by 43.3% of the participants, and similar findings were mentioned in the parallel study carried out by Saniasiaya et al., which demonstrated the link between COVID- 19 infection and dizziness and vertigo [15].

Anosmia has been an alerting symptom of COVID-19 by many [16]. In our study, less than half of the participants (45.5%) complained of anosmia/hyposmia: about one-fifth (19.9%) were sometimes exposed to it, 11.8% were usually exposed to it, and 13.8% were always exposed to anosmia/hyposmia. This percentage is similar to Borah et al.'s finding in which 44% of the participants had anosmia [17].

Upper airway and GERD symptoms were more prominent during the acute phase of the infection but were unremarkable in the long term. More than half (54%) had a sore throat during infection with COVID-19 but only 5.5% complained of it in the long term. Dysphagia was reported by 38.6% of the participants as an acute-phase presentation. However, the long-term outcome was not assessed. This percentage was higher than Adkins et al.'s finding of 16.1%, and this could be attributed to genetic host factors and environmental factors [18].

Hearing loss was reported in COVID-19 infection by Masalski et al. with a prevalence of 11-28% among his study participants [19]. In our study, it was much lower with an incidence of 4.8%. Interpretation of such findings seems difficult because it might reflect a wide range of hearing loss from mild conductive hearing loss to profound sensorineural hearing loss. However, we thought to keep this for future investigations and exploration.

Smoking habits were evaluated to have a significant correlation with epistaxis, while congestion/rhinorrhea was significantly higher among non-smokers. Similar findings were reported in the study carried out by Reh et al., in which smoking was linked with rhinosinusitis [20]. Otology-related symptoms were likewise differently correlated to smoking habits. Dizziness/vertigo was significantly higher among non-smokers while hearing loss was higher among smokers. This finding was consistent with the findings of Istrate et al. in which a positive correlation between smoking and hearing loss was demonstrated [21]. As for upper airway/GERD-related symptoms, except for heartburn, all were significantly higher among non-smokers.

The status of the disease was also correlated to symptoms; patients who stated they were in recovery but still have complications showed a significant correlation for nasal congestion/rhinorrhea as well as anosmia/hyposmia. Other rhinology symptoms were significantly correlated to the group of patients who had symptoms worsened with no recovery yet. Concerning upper airway/GERD-related symptoms, it was found that sore throat, loss of taste, and hoarseness of voice were higher among participants who had but still had complications while dysphagia and heartburn were higher among non-recovered participants with worsened symptoms. All the otology-related symptoms including ear pain/pressure, tinnitus, hearing loss, dizziness /vertigo, and ear fullness were found to be significantly higher among non-recovered participants with worsened symptoms.

## Conclusions

The most commonly reported initial symptom when they were diagnosed with COVID-19 was fever followed by headache. Interestingly, vertigo is a frequent long-term complication after a COVID-19 infection. Other otology-related symptoms include hearing loss and ear blockage, while rhinology-related symptoms were less frequent in the long term after the acute phase of the infection.

## References

[1] Hu B, Guo H, Zhou P, Shi ZL (2021). Characteristics of SARS-CoV-2 and COVID-19. Nat Rev Microbiol.

[2] Chen N, Zhou M, Dong X (2020). Epidemiological and clinical characteristics of 99 cases of 2019 novel coronavirus pneumonia in Wuhan, China: a descriptive study. Lancet.

[3] Bhatta S, Gandhi S, Saindani SJ, Ganesuni D, Ghanpur AD (2021). Otorhinolaryngological manifestations of coronavirus disease 2019: a prospective review of 600 patients. J Laryngol Otol.

[4] Alrusayyis D, Aljubran H, Alshaibani A (2022). Patterns of otorhinolaryngological manifestations of Covid-19: a longitudinal questionnaire-based prospective study in a tertiary hospital in Saudi Arabia. J Prim Care Community Health.

[5] Telmesani LM, Althomaly DH, Buohliqah LA (2022). Clinical otorhinolaryngological presentation of COVID-19 patients in Saudi Arabia: a multicenter study. Saudi Med J.

[6] (2022). Centers for Disease Control and Prevention. National Center for Immunization and Respiratory Diseases (NCIRD). 2019-2020 US Flu Season: Preliminary In-Season Burden Estimates. Estimates.

[7] Adly HM, AlJahdali IA, Garout MA, Khafagy AA, Saati AA, Saleh SA (2020). Correlation of COVID-19 pandemic with healthcare system response and prevention measures in Saudi Arabia. Int J Environ Res Public Health.

[8] Garout MA, Saleh SA, Adly HM (2022). Post-COVID-19 syndrome: assessment of short- and long-term post-recovery symptoms in recovered cases in Saudi Arabia. Infection.

[9] Khodeir MM, Shabana HA, Rasheed Z (2021). COVID-19: Post-recovery long-term symptoms among patients in Saudi Arabia. PLoS One.

[10] AlRadini FA, Alamri F, Aljahany MS (2022). Post-acute COVID-19 condition in Saudi Arabia: a national representative study. J Infect Public Health.

[11] Jabali MA, Alsabban AS, Bahakeem LM, Zwawy MA, Bagasi AT, Bagasi HT, Aldosary TA (2022). Persistent symptoms post-COVID-19: an observational study at King Abdulaziz Medical City, Jeddah, Saudi Arabia. Cureus.

[12] Samannodi M, Alwafi H, Naser AY (2022). Determinants of post-COVID-19 conditions among SARS-CoV-2-infected patients in Saudi Arabia: a web-based cross-sectional study. Diseases.

[13] Ng WH, Tipih T, Makoah NA (2021). Comorbidities in SARS-CoV-2 patients: a systematic review and meta-analysis. mBio.

[14] Amin MT, Hasan M, Bhuiya NM (2021). Prevalence of Covid-19 associated symptoms, their onset and duration, and variations among different groups of patients in Bangladesh. Front Public Health.

[15] Saniasiaya J, Kulasegarah J (2021). Dizziness and COVID-19. Ear Nose Throat J.

[16] Hopkins C, Surda P, Whitehead E, Kumar BN (2020). Early recovery following new onset anosmia during the COVID-19 pandemic - an observational cohort study. J Otolaryngol Head Neck Surg.

[17] Borah H, Das S, Goswami A (2022). Otorhinolaryngological manifestations and its management in COVID 19 patients. Indian J Otolaryngol Head Neck Surg.

[18] Adkins C, Takakura W, Spiegel BM, Lu M, Vera-Llonch M, Williams J, Almario CV (2020). Prevalence and characteristics of dysphagia based on a population-based survey. Clin Gastroenterol Hepatol.

[19] Masalski M, Morawski K (2020). Worldwide prevalence of hearing loss among smartphone users: cross-sectional study using a mobile-based app. J Med Internet Res.

[20] Reh DD, Higgins TS, Smith TL (2012). Impact of tobacco smoke on chronic rhinosinusitis: a review of the literature. Int Forum Allergy Rhinol.

[21] Istrate M, Hasbei-Popa M, Iliescu DA, Ghita AC, Ghita AM (2021). Effects of cigarette smoking on sensorineural hearing impairment and age related macular degeneration. Tob Prev Cessat.

